# Differences in Metabolic Profiles of Healthy Dogs Fed a High-Fat vs. a High-Starch Diet

**DOI:** 10.3389/fvets.2022.801863

**Published:** 2022-02-17

**Authors:** Yang Lyu, Daisy Liu, Patrick Nguyen, Iain Peters, Romy M. Heilmann, Veerle Fievez, Lieselot Y. Hemeryck, Myriam Hesta

**Affiliations:** ^1^ECAN Equine and Companion Animal Nutrition, Faculty of Veterinary Medicine, Ghent University, Merelbeke, Belgium; ^2^Nutrition, Physiopathology and Pharmacology Unit, National College of Veterinary Medicine, Food Science and Engineering, Nantes, France; ^3^SYNLAB VPG, Exeter Science Park, Exeter, United Kingdom; ^4^Gastrointestinal Laboratory, Department of Small Animal Clinical Sciences, College of Veterinary Medicine and Biomedical Sciences, Texas A&M University, College Station, TX, United States; ^5^Department of Animal Sciences and Aquatic Ecology, Faculty of Bioscience Engineering, Ghent, Belgium; ^6^Laboratory of Chemical Analysis, Faculty of Veterinary Medicine, Ghent University, Merelbeke, Belgium

**Keywords:** starch, fat, fecal metabolome, obesity, dogs

## Abstract

Obesity is a common problem in dogs and overconsumption of energy-rich foods is a key factor. This study compared the inflammatory response and fecal metabolome of dogs fed a high-fat vs. a high-starch diet. Ten healthy lean adult beagles were equally allocated into two groups in a cross-over design. Each group received two diets in which fat (horse fat) and starch (pregelatinized corn starch) were exchanged in an isocaloric way to compare high fat vs. high starch. There was a tendency to increase the glucose and glycine concentrations and the glucose/insulin ratio in the blood in dogs fed with the high-fat diet, whereas there was a decrease in the level of Non-esterified fatty acids and a tendency to decrease the alanine level in dogs fed with the high-starch diet. Untargeted analysis of the fecal metabolome revealed 10 annotated metabolites of interest, including L-methionine, which showed a higher abundance in dogs fed the high-starch diet. Five other metabolites were upregulated in dogs fed the high-fat diet, but could not be annotated. The obtained results indicate that a high-starch diet, compared to a high-fat diet, may promote lipid metabolism, anti-oxidative effects, protein biosynthesis and catabolism, mucosal barrier function, and immunomodulation in healthy lean dogs.

## Introduction

Obesity is one of the largest health challenges nowadays in dogs. Studies report a prevalence of canine overweight and obesity ranging from 34 to 60% (1–3). Obesity in dogs has been linked to not only a decreased vitality, emotional wellbeing, and longevity but also an increased risk of certain health issues such as insulin resistance, hypertension, cardiovascular disease, and osteoarthritis (4, 5). While multiple molecular mechanisms might link obesity to its complications, inflammation is a common feature that has been implicated in the pathophysiology of many obesity-associated disorders (6). Similarly to these findings in humans, it has been revealed that obese and overweight dogs showed a higher inflammatory state (7), as indicated by increased concentrations of serum interleukin-6 (IL-6) (7), C-reactive protein (CRP), and tumor necrosis factor alpha (TNF-α) (8).

The increase in adiposity is often attributed to high dietary fat intake (9). Many studies have shown that high-fat diets (>30% of energy from fat) can easily induce obesity in humans (10, 11), mice (12, 13), and dogs (14, 15). When the average amount of fat in the diet increases, the incidence of obesity also rises (11, 12). Furthermore, in mice and humans, consumption of high-fat diets leads to alterations in the composition and function of the gut microbiota, promoting metabolic endotoxemia and triggering an inflammatory response (13, 16). In dogs, feeding a high-fat diet is associated with insulin resistance (17), reduced brain insulin transport (14), decreased microbiota α-diversity (15), and reduced abundance of *Prevotella, Solobacterium*, and *Coprobacillus* (18). However, the majority of studies on the intake and possible adverse health effects of a high-fat diet in dogs focused on increased inflammation and alterations in the gut microbiome, but no study so far has investigated the effect of a high-fat diet on the gut metabolome.

Starch is the most abundant dietary nutrient globally, and provides energy to a rapidly growing human population (19), and is also a main nutritional source for pet dogs (20). However, this highly digestible energy source could also lead to nutrition-related health problems. Studies have suggested that typical starch-rich diets can also contribute to obesity (19, 21), hyperglycaemia (21), pathogenesis and difficulty of managing type 2 diabetes mellitus (22), as well as cardiovascular disease (23). In dogs, nutritional research on dietary starch mainly concerns its digestibility and fermentation, but not the effect of high-starch diets on metabolic changes and inflammatory responses.

Metabolomics is the study of all small molecules detectable in a biological sample. It provides information on subclinical metabolic alterations associated with (patho)physiological changes and disease outcomes (24). Metabolome analysis has revealed previously unknown alterations in amino acid, lipid, and carbohydrate metabolism across species, with underlying links to several conditions like obesity, inflammation, and oxidative stress (25). Metabolomics is rather emerging in canine nutrition, with current research being limited to study the metabolomic profile in the healthy vs. obese or overweight dogs (26), and in dogs fed with different protein levels (27), with far more pending to be explored. The present study aimed to compare the inflammatory status and fecal metabolome of lean dogs fed a high-fat vs. a high-starch diet, providing new insights and basis for a theoretical framework for high-fat vs. high-starch induced metabolic and inflammatory effects in relation to obesity.

## Materials and Methods

### Animals and Experimental Design

Ten healthy adult research beagles of ideal body weight (BW) and condition (4 intact females, 3 intact males and 3 neutered males; 4.2 ± 2.6 y; 10.5 ± 1.2 kg; body condition score (BCS) 4–5/9) were equally allocated into two groups in a cross-over study design with two periods of 6 weeks each. All dogs were housed individually and under a 12-h light and 12-dark cycle with a room temperature of 17°C. Prior to the study, a commercial standard diet (Hill's Science Plan Advanced Fitness; Hill's Pet Nutrition, Inc., Topeka, KS, USA) mixed with experimental diets was fed to the dogs for 1 week adaption −75% standard with 25% experimental diets for 3 days, 50% of each for 2 days, and 75% experimental with 25% standard diets for 2 days. During the first period of the study (P1), five dogs in group A were fed a high-starch (HS; pregelatinized corn starch; ~63.5% carbohydrate and 9.4% fat) diet and five dogs in group B were fed a high-fat (HF; horse fat; ~12.9% carbohydrate and 46.9% fat) diet. After P1, a mixture of two experimental diets was fed to the dogs for one-week transition–group A: 75% HS with 25% HF diet for 3 days, 50% of each for 2 days, and 25% HS with 75% HF for 2 days; same proportion but reverse diets for group B. Experimental diets were then completely switched during the second period of the study (P2).

Diets were formulated to be isonitrogenous on energy basis, so that for a given energy allocation the protein intake was similar regardless of the diet. The formulation of both diets is presented in Table 1. The HF and HS diet contained 18.2 g crude fat, 13.9 g crude protein, and 5.0 g nitrogen-free extract per MJ, and 5.2 g crude fat, 13.1 g crude protein, and 35.0 g nitrogen-free extract per MJ, respectively. Dogs were fed once a day at 10:00 a.m., and had free access to water. Body weight and BCS were assessed weekly. Food intake was recorded daily, and the amount was adjusted weekly to maintain a stable body weight, if needed.

**Table 1:** Formulation of the diets (g/kg).

**Item**	**High Starch diet (HS)**	**High Fat diet (HF)**
Horse hearts	701.5	827.6
Corn starch (pregelatinized)	287.6	0.0
Corn oil	3.9	4.6
Horse fat	1.4	161.4
Premix	5.6	6.4
KJ/100g Dry matter	1,811	2,579

All samples were collected at the end of each study period. Fasting blood samples (~30 mL) were drawn from the jugular vein. An aliquot of ~4 mL was collected in PAXgene Blood RNA tubes (PreAnalytiX GmbH, Erembodegem, Belgium) to analyse mRNA expression of TLR-4, CD14, IL-10, IL-18, IL-1B, IL-1RA, IL-8, and TNF-α. Serum and plasma for assessing mRNA expression, acylcarnitine and amino acid profiles were obtained by centrifuging blood at 2000 x g for 15 min at 4°C, which was stored at −20°C until analysis. Fresh fecal samples (~10 g) were collected within 10 min after spontaneous defaecation. The samples were scored for fecal consistency (1 = watery liquid feces that can be poured; 2 = soft, unformed stool that assumes the shape of the recipient; 3 = soft, formed, moist stool; 4 = hard, formed, dry stool; 5 = hard, dry stool), and fecal pH was measured with a portable pH meter (Hanna Instruments Ltd., Temse, Belgium). An aliquot of ± 2 g was placed into a sterile plastic tube, frozen immediately on dry ice, lyophilized as soon as possible, and stored at −80°C in preparation of metabolomic analysis. The remainder of the fecal sample was stored at −20°C for chemical analyses.

### Analytical Methods

Body composition was determined by the deuterium dilution method using Fourier-transform infrared spectroscopy as described by (28).

Proximate analysis was performed on the diets using standard methods, ISO 1442:1997 for dry matter, ISO 936:1998 for crude ash, Kjeldahl nitrogen (6.25 × N, ISO 5983–1, 2005) for dietary crude protein, and ISO 5498:1981 for crude fiber. Nitrogen-free extract was calculated by subtracting crude ash, crude protein, crude fat, and crude fiber from the dry matter content. A Total Dietary Fiber Assay Kit (Sigma–Aldrich Co., Overijse, Belgium) was used to determine total dietary fiber and insoluble dietary fiber using procedures based on a combination of enzymatic and gravimetric methods (29). Soluble dietary fiber was calculated by subtracting insoluble dietary fiber from total dietary fiber.

Serum concentrations of glucose, triglyceride, cholesterol, and total protein were determined using the Architect C16000 analyser (Abbott Max-Planck-Ring, Wiesbaden, Germany). Fibrinogen concentration was determined using the Sysmex CS-5100 analyser (Siemens Healthcare Diagnostics Products GmbH, Marburg, Germany), insulin concentration was determined by a commercially available kit (INS-Irma, DIAsource ImmunoAssays S.A., Louvain-la-Neuve, Belgium), and the insulin-to-glucose ratio was calculated as described in German et al., (30) to assess insulin sensitivity. Serum leptin concentration was measured using a validated, commercially available canine ELISA kit (Millipore Corp., Billerica, MA, USA) following the manufacturer's instructions. Serum Non-esterified fatty acids (NEFA) concentrations were analyzed by spectrophotometry (EZ Read 400 Microplate Reader, Biochrom Ltd., Cambridge, United Kingdom). Free carnitine, acylcarnitine and amino acid profiles were determined on lithium-heparin plasma by quantitative electrospray tandem mass spectrometry as previously described (31, 32). Blood lipopolysaccharides (LPS) concentrations were determined using a kinetic turbidimetric Limulus amoebocyte lysate (LAL) assay.

S100A12 concentration in serum and feces was determined by a species-specific ELISA (33). Fecal short-chain fatty acid [SCFA; i.e., acetate, propionate, butyrate, iso-butyrate, iso-valerate], and NH_3_ concentrations were determined first by extracting samples with 10% formic acid, containing 1 mg/ml 2-ethyl butyric acid as internal standard (3 g sample + 15 ml extraction fluid; shake for 5 min, centrifugate, and filtrate). The determination of respectively the volatile fatty acids and ammonia was carried out using gas chromatography as previously described (34, 35).

### mRNA Expression

Total RNA was isolated from the PAXgene tubes using the PAXgene blood RNA kit (Qiagen, Manchester, UK) according to the manufacturer's instructions. RNA concentration was measured using the Qubit RNA Assay Kit (Invitrogen, Paisley, Scotland). Primers and probes for the assay were designed using Primer 3 (www.genome.wi.mit.edu/cgibin/primer/primer3_www.cgi.) and M-Fold using the canine specific GenBank sequence for IL-1β (EU249360) and IL-1α (AF216526) as described previously (36). The assays for the 3 housekeeper genes (succinate dehydrogenase complex, subunit A [SDHA], TATA box binding protein [TBP], and tyrosine 3-monooxygenase/tryptophan 5-monooxygenase activation protein zeta polypeptide [YWAZ]) and the remaining genes (Supplementary Table 1) were the same as those used previously (37).

Synthesis of cDNA was carried out using the ImProm II Reverse Transcription System (Promega Corporation) with 500 ng of total RNA in a final volume of 20 μL. Quantitative PCR (qPCR) was performed using GoTaq Colorless Master Mix (Promega). Gene specific amplification was performed using 0.2 μM of each primer, 0.1 μM of the probe, ROX (1:5000, Invitrogen) and 5 μl of diluted cDNA in a final volume of 25 μl. Sample incubations were performed in an MxPro 3005P (Agilent) at 95°C for 2 min and then 45 cycles of 95°C for 10 sec and 60°C for 30 sec during which the fluorescence data were collected. Threshold values (Ct) for the samples were calculated using the MxPro qPCR software 4.1 (Agilent Technologies Co., Santa Clara, CA, USA). Relative copy number expression values were calculated for each sample and normalized against the housekeeper gene results using the qBase applet for Microsoft Excel (http://medgen.ugent.be/qbase/) as described by Vandesompele et al. (38).

### Untargeted Metabolomics Analysis

Freeze-dried fecal samples were subjected to generic extraction as optimized and described previously (39). Analysis of extracted fecal samples was performed using a Dionex UltiMate 3000 XRS UHPLC system (Thermo Fisher Scientific, San José, CA, USA) coupled to a Q-Exactive™ bench top Quadrupole-Orbitrap HRMS (Thermo Fisher Scientific, San José, CA, USA) (39, 40). Calibration of the Q-Exactive HRMS system was performed according to the instructions of the manufacturer. Internal (each 10 samples) and external QC (quality control) samples (pool of samples made from aliquots of the study samples) were analyzed prior to and after analysis of the samples to stabilize the system and monitor (and if needed, correct for) instrumental drift. Samples were analyzed in 1 batch, in a randomized order.

Raw data was Pre-processed using Sieve™ 2.1 (Thermo Fisher Scientific, San José, USA), as described by De Paepe et al., (40). Simca™ 13 (Umetrics AB, Umeå, Sweden) was used for multivariate statistical data processing. PCA-X and OPLS-DA modeling were performed following logarithmic data transformation and Pareto scaling, with further validation assessed by assessment of R2 and Q2 goodness (>0.5), permutation testing (*n* = 100) and cross-validated analysis of variance (CV-ANOVA, *p* < 0.05). Discriminative/predictive ions were selected based on their eccentric position in the S-plot [*p* (corrected) > |0.5|] and a Variable Importance in Projection-score (VIP score) >2. Prediction of chemical formula was based on accurate mass and the full scan spectrum using Xcalibur^TM^; i.e., obtained through calculation and evaluation of isotopic signature (carbon and sulfur) and allowing a max mass deviation of 5 ppm. Putative identification was achieved using the Human Metabolome Database (HMDB), PubChem and Kyoto Encyclopedia of Genes and Genomes (KEGG) databases (freely available online). A heatmap with dendrogram was generated using TBtools software (https://github.com/CJ-Chen/TBtools) to illustrate metabolite abundances and sample clustering.

### Statistical Analyses

The metabolomic data were processed as described above. The effect of diets, periods and their interaction were analyzed by two-way ANOVA using MetaboAnalyst 3.0 software (McGill University, Canada).

To evaluate the effect of both the diet and the two-period dietary exchange, the remainder of the data was analyzed by Wilcoxon-Mann–Whitney test with diet, period and diet^*^period as factors and dog as random effect. These analyses were processed by R version 3.1.0 (The R Foundation for Statistical Computing) using the Coin package (version 1.0–23). Summary statistics were expressed as mean values ± SD. A *p*-value of < 0.05 was considered statistically significant and a *p*-value < 0.10 was considered as a significant trend. All *p*-values were corrected by the false discovery rate.

## Results

### Food Intake and Body Composition

All dogs remained healthy throughout the study. All diets were well tolerated and did not affect the dogs' food intake. Daily energy intakes did not differ between diets. There was no significant diet and period effect on BW and BCS at the end of each study period. Furthermore, neither diet nor period significantly affected the dogs' absolute and relative body fat mass.

### Fecal Parameters

There was no significant effect of diet and period on the fecal concentration of NH3, acetate, propionate, butyrate, iso-butyrate, and iso-valerate. The diets also did not affect the fecal S100A12 concentrations, and no significant effect was observed on the fecal score and pH. These results are summarized in Supplementary Table 2.

### Blood Parameters

Significant findings in blood parameters are presented in Table 2, while blood parameters which did not significantly differ between diets or periods are available in Supplementary Table 3. A significant diet effect (*p* = 0.041) was observed for the Pre-prandial NEFA concentration, with dogs being fed the high-starch diet showing a higher level of NEFA than dogs being fed the high-fat diet. Diet × period trends were observed for the plasma concentration of glycine (*p* = 0.077) and tyrosine (*p* = 0.058). Additionally, significant trends according to diet were observed for the plasma concentration of glucose (GLU; *p* = 0.054), glycine (Gly; *p* = 0.094), alanine (Ala; *p* = 0.089), and the ratio of glucose to insulin (GLU/INS; *p* = 0.063). More specifically, dogs on the high-fat diet displayed a trend for a decreased Ala concentration and an increased GLU concentration and GLU/INS ratio compared to dogs on the high-starch diet. Significant effects of period were also observed for the GLU/INS ratio (*p* = 0.036) and NEFA concentration (*p* = 0.042).

**Table 2:** Blood parameters (significant findings; *n* = 5).

**Item**	**HS**	**HF**	**Period 1**	**Period 2**	***p*** **value**		
					**Diet**	**Period**	Diet^*^Period
GLU (μM)	78.40 ± 6.28	85.70 ± 5.21	83.10 ± 5.99	81.00 ± 7.60	0.054	0.324	0.134
GLU/INS	16.25 ± 5.54	46.46 ± 59.15	45.24 ± 59.59	17.48 ± 7.53	0.063	0.036	0.145
Gly (μM)	190.1 ± 24.12	186.9 ± 37.32	201.0 ± 33.47	176.0 ± 22.61	0.094	0.220	0.077
Ala (μM)	358.6 ± 118.5	239.5 ± 44.27	335.4 ± 129.1	262.8 ± 65.54	0.089	0.159	0.347
NEFA (μM)	1.13 ± 0.25	0.94 ± 0.23	1.13 ± 0.31	0.95 ± 0.15	0.041	0.042	0.108
Phe (μM)	85.11 ± 18.94	84.04 ± 16.98	80.64 ± 11.61	88.51 ± 21.88	0.139	0.077	0.120
Tyr (μM)	54.87 ± 8.93	51.45 ± 10.59	53.82 ± 8.63	52.49 ± 11.09	0.113	0.085	0.058

*GLU, glucose; INS, insulin; Gly, glycine; Ala, alanine; NEFA, Non-esterified fatty acid; Phe, phenylalanine; Tyr, tyrosine*.

No significant dietary effect or trend was observed for LPS concentration, acylcarnitine profiles and mRNA expression levels in the blood (Supplementary Table 3).

### Fecal Metabolome

A total of 4391 and 1934 ions were obtained in the positive and negative ionization mode, respectively. PCA-X score plots revealed good clustering of fecal samples according to diet in both positive and negative ionization mode (Supplementary Figure 1), as well as good clustering of QC samples. The characteristics of the OPLS-DA model (Supplementary Figure 2) were good to excellent: R^2^Y = 0.884 and Q^2^ = 0.661 for the “positive” model and R^2^Y = 0.861 and Q^2^ = 0.670 for the “negative” model, also obtaining successful cross-validation (CV-ANOVA with *p* <0.01), as well as a valid permutation test. A total of 15 fat/starch associated metabolites could be retained (Supplementary Figure 3), with 5 unidentified metabolites and 9 putatively annotated metabolites. The identity of one metabolite marker; i.e., L-methionine, was confirmed by means of an analytical standard. An overview of the (characteristics of the) retrieved discriminative metabolites is presented in Table 3.

**Table 3:** Overview of characteristics of L-methionine, putatively annotated and unidentified metabolite markers.

**Compound n**°****	Putative identification^*^	**ID level**	**Formula**	***m/z***	**ppm**	**RT**	**Ionization mode**	**VIP score**	**Reference**
1	Unidentified_1	–	–	223.1234	/	0.91	H^+^	2.485	–
2	Unidentified_2	–	–	415.2172	/	0.97	H^+^	2.373	–
3	L-Methionine	1	C_5_H_11_NO_2_S	150.0580	2.04	1.57	H^+^	2.996	HMDB
4	Glycyl-Valine/Glycyl-Norvaline/Valyl-Glycine/L-Theanine/N-acetylornithine	4	C_7_H_14_N_2_O_3_	173.0931	0.44	1.65	H^−^	3.816	HMDB
5	Unidentified_3	–	–	356.1478	/	1.75	H^−^	2.481	–
6	Spermic acid 2/(Iso)leucyl-threonine/ Threoninyl-(Iso)leucine	4	C_10_H_20_N_2_O_4_	231.1357	2.72	2.22	H^−^	2.321	HMDB
7	L-Lysopine/(Iso)leucyl-serine/seryl-(Iso)leucine/Valyl-Threonine/Threoninyl-Valine	4	C_9_H_18_N_2_O_4_	219.1333	2.89	2.32	H^+^	3.379	HMDB
8	Valyl-valine	4	C_10_H_20_N_2_O_3_	217.1542	2.34	4.24	H^+^	3.160	HMDB
9	Spermic acid 2/(Iso)leucyl-threonine/ Threoninyl-(Iso)leucine	4	C_10_H_20_N_2_O_4_	231.1358	3.33	4.88	H^−^	3.526	HMDB
10	Spermic acid 2/(Iso)leucyl-threonine/ Threoninyl-(Iso)leucine	4	C_10_H_20_N_2_O_4_	231.1357	3.03	5.16	H^−^	4.124	HMDB
11	(Iso)leucyl-valine/Valyl-(Iso)leucine	4	C_11_H_22_N_2_O_3_	229.1566	3.47	5.54	H^−^	3.426	HMDB
12	(Iso)leucyl-Threoninyl-Valine	4	C_15_H_29_N_3_O_5_	332.2173	2.01	6.88	H^+^	3.430	PubChem
13	(Iso)leucyl-(Iso)leucine	4	C_12_H_24_N_2_O_3_	243.1722	3.39	6.98	H^−^	3.553	HMDB
14	Unidentified_4	–	–	283.1200	/	7.12	H^−^	3.096	–
15	Unidentified_5	–	–	263.0809	/	9.17	H^+^	2.466	–

*ID level, metabolite identification level according to Sumner et al. (41); ppm, absolute difference theoretical vs. detected m/z; RT, retention time (min.); VIP, variable importance in projection-score*.

* *IUPAC names are provided in Supplementary Table 5*.

The results obtained for investigating the interaction among diet, period and their interaction are summarized in Supplementary Table 4. All 15 metabolites were significantly influenced by the two diets, while none of them was significantly influenced by the study periods. One unidentified metabolite (Unidentified_1) was subject to the interaction between diet and period.

**Figure 1:**
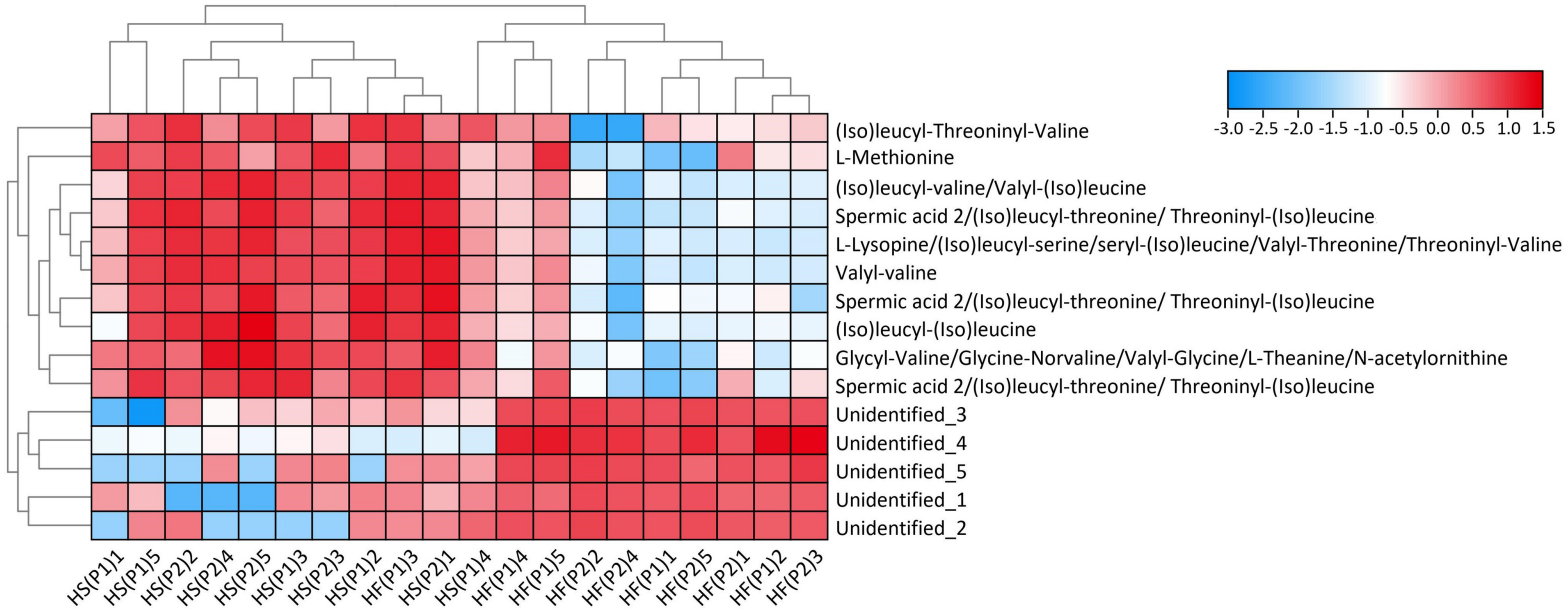
Heatmap with cluster dendrogram of retrieved discriminative metabolites. Colors represent log-transformed peak intensity of metabolites. HF = high-fat diet, HS = high-starch diet, P1 = study period 1 and P2 = study period 2 for 5 dogs per group.

Normalized abundances of metabolites discriminating for the high-fat vs. high-starch diet are presented in a heatmap in Figure 1. Clear clustering of the samples according to diets was observed (except for one sample HF(P1)3). Moreover, dogs fed the high-fat diet displayed a significantly higher abundance of 5 Non-annotated molecules, whereas dogs fed the high-starch diet displayed a significantly higher abundance of L-methionine, 6 molecules tentatively identified as (iso)leucyl-threoninyl-valine; (iso)leucyl-(iso)valine or (iso)valyl-(iso)leucine; L-lysopine or (iso)leucyl-serine/seryl-(iso)leucine or valyl-threonine or threoninyl-valine; glycyl-valine or valyl-glycine or gly-norvaline or L-theanine or N-acetylornithine; valyl-valine; and (iso)leucyl-(iso)leucine, as well as 3 molecules putatively identified as spermic acid 2 or (iso)leucyl-threonine or threoninyl-(iso)leucine.

## Discussion

Most nutritional studies on the inflammatory response and metabolism in dogs have focused on the effects of protein (27, 42, 43). However, there is less information concerning dietary fat and nonfibrous carbohydrates, which are the main energy-delivering nutrients in traditional dog food (44). Health issues, particularly obesity-related problems, associated with diets rich in fat and starch, have received increasing attention in recent canine studies (14, 18, 45). For the first time, the present study investigated the inflammatory response and fecal metabolome in healthy lean dogs fed a high-fat or a high-starch diet.

### No Effect on Inflammatory Related mRNA Expression

Obesity is characterized as a state of low-grade systemic inflammation, in which many inflammatory cytokines appear to play a role. They might also be linked to co-morbidities of obesity (46). Recent evidence suggests that a high-fat diet (60% fat) can induce obesity and exacerbate obesity-related inflammation and metabolic disorders in mice (47, 48). Moreover, gut inflammation is more severe in obesity-prone rats compared to obesity-resistant rats when both were fed a high-fat diet (45.3% fat) (49). Inflammation was also selectively more marked in the short-term high-fat-fed mice (60% fat) (50), which might depend on the level of weight gain. However, no significant changes were noted in the inflammatory related mRNA expression in this study. Overall, this result is not surprising as the dogs were fed to keep an ideal body condition and body weight. In fact, in our study, the two diets were isoenergetically exchanged, with the high-fat diet not exceeding the safe upper limit for dogs (70% for adult maintenance) (51). Next to this, except for the species differences and susceptibility to obesity, as genetically modified mice are more prone to developing morbid obesity (52), the discrepancy may be ascribed to the study's limited experimental duration, tested parameters or sample types. The study was not designed to investigate potential long-term effects. Tissue samples could not be collected, or more invasive parameters tested (e.g., inflammatory markers in fat or intestinal mucosal) in the dogs due to ethical reasons. Also, differences in the fat source and different levels of dietary fat intake between dogs and mice studies might also influence the experimental results. Therefore, future research investigations need to evaluate an extended set of inflammatory parameters under a variety of experimental conditions to unravel the inflammatory response of dogs fed a high-fat vs. a high-starch diet.

### Observed Metabolic Effects in Blood

Vast differences were found in the metabolic profiles in blood our study. When dogs were fed with the high-fat diet, there is a tendency to increase glucose concentration and the ratio of glucose to insulin in the blood. This result suggests that the high-fat diet can affect glucose metabolism in healthy dogs. Previously, a high-starch diet (43% energy from starch and 26% from fat) has also been shown to increase glucose and insulin concentrations in healthy dogs compared to isoenergetic low- (12% energy from starch and 40% from fat) and moderate-starch diets (30% energy from starch and 34% from fat) (53). However, both fat and protein contents were adjusted to formulate the low- and moderate-starch diets, making it difficult to evaluate the effect of only fat in this study (53). Another study did not find any significantly changes in plasma glucose and insulin concentrations in dogs fed with a high-fat diet (63% energy from fat and 12% from starch) at two energetic intake levels (100% and 150% of maintenance energy requirements [MER]). Insulin sensitivity, however, was lower in the dogs of 150% MER group, which also had higher BW and BCS (15). The present study was not designed to analyse the effect on insulin sensitivity as only Pre-prandial samples were drawn. Thus, future research is needed to explore the possibility of such an effect. In the present study, the high-starch diet was associated with different metabolic effects compared to the high-fat diet in healthy lean dogs, as indicated by the increased NEFA concentration and a trend for increased Ala concentrations in the blood. Previous studies have reported that the increased Ala and NEFA levels are associated with increased glucose and lipid metabolism. Alanine is the key protein-derived gluconeogenic precursor, and plasma NEFA arise mainly from hydrolysis of triacylglycerol within the adipocyte (54, 55). This result suggests that compared to a high-fat diet, a high-starch intake could influence host glucose and lipid metabolism in healthy dogs. Future studies are needed to further investigate the metabolic effects of high-starch in exchange for high-fat diets.

### Observed Metabolic Effects in Feces

Metabolomic research in dogs is still at a preliminary stage, with a limited number of published studies. Most of those studies focus on studying the metabolome in the context of disease or following dietary supplementation (56–59). However, the current study investigated shifts in the fecal metabolome of dogs on a high-starch vs. high-fat diet for the very first time. Polar metabolomics revealed a distinctively different fecal metabolomic profile in dogs fed a high starch vs. high fat diet. Specifically, the high-starch diet increased the abundance of L-methionine, and several molecules that were tentatively annotated as (iso)leucyl-threoninyl-valine, (Iso)leucyl-(Iso)valine/(Iso)valyl-(Iso)leucine, L-lysopine/(iso)leucyl-serine/seryl-(iso)leucine/ valyl-threonine/threoninyl-valine, valyl-valine, (iso)leucyl-(iso)leucine, glycyl-valine/valyl-glycine/gly-norvaline/L-theanine/N-acetylornithine, and spermic acid 2/(iso)leucyl-threonine/threoninyl-(iso)leucine.

Of the (putatively) annotated fecal metabolites, several are known to exert anti-oxidative and immunomodulatory effects. Methionine for example plays a critical role in the metabolism and health of many species, including dogs (60), as it is an essential amino acid involved in protein as well as aminoacyl-tRNA biosynthesis. Interestingly, there is accumulating evidence for aminoacyl-tRNA synthetases being involved in a wide range of physiological and pathological processes, including different types of immune responses (61). Recent research has furthermore demonstrated that methionine intervenes in lipid metabolism and anti-oxidation (61, 62).

Based on accurate mass, we assume compound n°4 may either be a dipeptide, L-theanine or N-acetylornithine, although confident identification could not be achieved. L-theanine is an L-glutamate and L-glutamine analog. Interestingly, glutamine analogs are known to improve intestinal mucosal repair function (63), whilst recent studies furthermore reported that L-theanine supplement affects intestinal mucosal immunity by regulating SCFA metabolism in rats (64) and broilers (65). These studies implied that increased abundance of L-theanine in this study may have beneficial effects on intestinal reparation and immune. N-Acetylornithine is an intermediate in the enzymatic biosynthesis of the amino acid L-arginine from L-glutamate (66), which involves protein synthesis, anti-oxidative and immunomodulatory effects as well as e.g., improved mucosal barrier function (67).

Besides compound *n*° 4, eight other marker molecules (*n*° 6–13) were also putatively identified as di- or tripeptides. Di- or tripeptides are incomplete breakdown products of protein digestion or intermediates in protein catabolism (68).

For compounds n° 6, 9 and 10, alternative tentative annotations include spermic acid 2, and for compound n° 7, putative identification as L-lysopine is a possibility besides being a dipeptide. Spermic acid 2 is an metabolite of putrescine and spermine (https://contaminantdb.ca/contaminants/CHEM041097, ContaminantDB, McGill University, Canada), which are produced by the collective microbiome (69). Putrescine and spermine are required for several physiological functions including protein synthesis, cell growth and differentiation (70), and spermine is furthermore known to supress inflammation (71). Increased spermic acid levels in this study thus implied the improvement of these roles by the high-starch diet. Regarding L-lysopine, a marker molecule with the same accurate mass was previously detected and putatively annotated in healthy suckling piglets (72). More specifically, the tentatively annotated L-lysopine was downregulated (*p* > 0.05) in piglet plasma following supplementation with an additional 0.12% methionine in the basal diet of sows during late gestation and lactation. Methionine supplementation showed a positive effect on piglet growth performance, which was hypothesized to be due to an increased antioxidant capacity of the piglets. This does not align with the proposed anti-oxidative effects of the high-starch vs. high-fat diet in this study since the putatively annotated L-lysopine molecule was higher following the intake of the high-starch vs. high-fat diet. According to HMDB, L-lysopine originates from food (or feed) (73), but there is very limited knowledge on spermic acid 2 and L-lysopine either dogs or any other species. Therefore, these findings warrant further investigation.

Five metabolite markers were upregulated following feeding of the high-fat diet compared to the high-starch diet. For compound n° 14 (“Unidentified_4”), a potential match with karalicin was retrieved in the PubChem database. There is however no existing prior knowledge about this compound either in any species, although it has been observed that it can produced by certain bacteria (74). Another potential HMDB match for compound n° 14 is 2-Phenylethyl beta-D-glucopyranoside (C14H20O6). This compound has previously been detected in caraway and citrus (73), but it is unclear whether this compound could therefore also present in the dog feed. Due to these uncertainties, compound n° 14 was not annotated as either karalicin or 2-Phenylethyl beta-D-glucopyranoside. Overall, metabolite identification is a major bottleneck in metabolomics research (75), and even more so for studies in dogs since there is no existing dog metabolome database.

### General Discussion

In summary, no difference was found in the inflammatory response in dogs fed with a high-starch vs. a high-fat diet, whereas different metabolic profiles were observed for the two diets. The high-starch diet in this study might be associated with several effects that indicated by the altered metabolic profiles, including protein biosynthesis, lipid metabolism, as well as exert anti-oxidation and/or immunomodulation. Future studies should encompass investigation of both short and long-term effects of high-starch in exchange for high-fat diets furthermore taking into account the source and level of fat intake. Moreover, in order to better understand the link between fecal metabolome and host metabolism, the analysis of microbiome is warranted in future studies, as well as multiple-omics analyses such as proteomics and lipidomics, which would enable studying the formation and/or degradation of proteins and the more a polar fraction of the metabolome. Lastly, it should be noted that Beagles in breeds are more prone to developing obesity (76) and therefore, follow-up studies should furthermore explore the effect of a high-fat vs. high-starch diet in relation to obesity and related problems, not only in Beagles, but also in other dog breeds, and in different age stages.

## Conclusion

Inflammatory and metabolic responses of dogs fed a high-fat and high-starch diet were evaluated in the present study. The inflammatory response did not differ between the two diets. The high-starch diet was associated with increased blood NEFA level, a tendance for increased blood Ala level and showed a profound impact on the fecal metabolomic profile with alterations of the abundance of 15 fecal metabolites including methionine, the high-fat dietary intake was associated with a trend to for increase the glucose concentration, and the glucose/insulin ratio in the blood and significantly increase in the abundance of 5 other metabolites. These alterations might be linked to promotion of lipid metabolism, anti-oxidative effects, protein biosynthesis and catabolism, mucosal barrier function and immunomodulation in healthy dogs.

## Data Availability Statement

The raw data supporting the conclusions of this article can be found in online repositories (Figshare, https://doi.org/10.6084/m9.figshare.17185376.v1).

## Ethics Statement

The animal study was reviewed and approved by Ethical Committee of the Faculty of Veterinary Medicine, Ghent University, Belgium.

## Author Contributions

YL analyzed and interpreted the data and drafted the first version of the manuscript. DL performed the feeding trial and collected the samples. PN analyzed the proximate analysis. RH analyzed the S100A12 concentrations. VF analyzed fecal SCFA, BCFA, and NH3 concentrations. LH performed the metabolomic analysis and was a major contributor in writing the manuscript. MH was the project administration who conceived and designed the experiment. All authors read and approved the final manuscript.

## Funding

This study was supported by the Morris Animal Foundation (D13CA-405).

## Conflict of Interest

IP was employed by the company SYNLAB VPG. The remaining authors declare that the research was conducted in the absence of any commercial or financial relationships that could be construed as a potential conflict of interest.

## Publisher's Note

All claims expressed in this article are solely those of the authors and do not necessarily represent those of their affiliated organizations, or those of the publisher, the editors and the reviewers. Any product that may be evaluated in this article, or claim that may be made by its manufacturer, is not guaranteed or endorsed by the publisher.

## References

[1] McGreevy PD Thomson PC Pride C Fawcett A Grassi T Jones B. Prevalence of obesity in dogs examined by Australian veterinary practices and the risk factors involved. Vet Rec. (2005) 156:695–702. 10.1136/vr.156.22.695 15923551

[2] Lund EM Armstrong PJ Kirk CA Klausner JS. Prevalence and risk factors for obesity in adult dogs from private US veterinary practices. Int J Appl Res Vet Med. (2006) 4:177.

[3] Diez M Picavet P Ricci R Dequenne M Renard M Bongartz A. Health screening to identify opportunities to improve preventive medicine in cats and dogs. J Small Anim Pract. (2015) 56:463–9. 10.1111/jsap.12365 25958785

[4] German AJ Holden SL Wiseman-Orr ML Reid J Nolan AM Biourge V. Quality of life is reduced in obese dogs but improves after successful weight loss. Vet J. (2012) 192:428–34. 10.1016/j.tvjl.2011.09.015 22075257

[5] Kealy RD Lawler DF Ballam JM Mantz SL Biery DN Greeley EH. Effects of diet restriction on life span and age-related changes in dogs. J Am Vet Med Assoc. (2002) 220:1315–20. 10.2460/javma.2002.220.1315 11991408

[6] Ferrante AW Jr. Obesity-induced inflammation: a metabolic dialogue in the language of inflammation. J Intern Med. (2007) 262:408–14. 10.1111/j.1365-2796.2007.01852.x 17875176

[7] Freeman LM Laflamme DP Michel KE. Comparison of adipokine concentrations and markers of inflammation in obese versus lean dogs. Intern J Appl Res Vet Med. (2009) 7:196–205.

[8] Rafaj RB Kules J Marinculic A Tvarijonaviciute A Ceron J Mihaljevic Z. Plasma markers of inflammation and hemostatic and endothelial activity in naturally overweight and obese dogs. BMC Vet Res. (2016) 13:13. 10.1186/s12917-016-0929-8 28061787 PMC5219720

[9] Hill JO Melanson EL Wyatt HT. Dietary fat intake and regulation of energy balance: implications for obesity. J Nutr. (2000) 130:284S−8S. 10.1093/jn/130.2.284S 10721889

[10] French S Robinson T. Fats and food intake. Curr Opin Clin Nutr Metab Care. (2003) 6:629–34. 10.1097/00075197-200311000-00004 14557792

[11] George V Tremblay A Despres JP Leblanc C Bouchard C. Effect of dietary fat content on total and regional adiposity in men and women. Int J Obes. (1990) 14:1085–94. 2086500

[12] Tucker LA Kano MJ. Dietary fat and body fat: a multivariate study of 205 adult females. Am J Clin Nutr. (1992) 56:616–22. 10.1093/ajcn/56.4.616 1414959

[13] Parks BW Nam E Org E Kostem E Norheim F Hui ST. Genetic control of obesity and gut microbiota composition in response to high-fat, high-sucrose diet in mice. Cell Metab. (2013) 17:141–152. 10.1016/j.cmet.2012.12.007 23312289 PMC3545283

[14] Kaiyala KJ Prigeon RL Kahn SE Woods SC Schwartz MW. Obesity induced by a high-fat diet is associated with reduced brain insulin transport in dogs. Diabetes. (2000) 49:1525–33. 10.2337/diabetes.49.9.1525 10969837

[15] Moinard A Payen C Ouguerram K Andre A Hernandez J Drut A. Effects of high-fat diet at two energetic levels on fecal microbiota, colonic barrier, and metabolic parameters in dogs. Front Vet Sci. (2020) 7:566282. 10.3389/fvets.2020.566282 33102570 PMC7545960

[16] Cotillard A Kennedy SP Kong LC Prifti E Pons N Le Chatelier E. Dietary intervention impact on gut microbial gene richness. Nature. (2013) 500:585–8. 10.1038/nature12480 23985875

[17] Rocchini AP Marker P Cervenka T. Time course of insulin resistance associated with feeding dogs a high-fat diet. Am J Physiol Endocrinol Metab. (1997) 272:E147–54. 10.1152/ajpendo.1997.272.1.E147 9038864

[18] Schauf S de la Fuente G Newbold CJ Salas-Mani A Torre C Abecia L. Effect of dietary fat to starch content on fecal microbiota composition and activity in dogs. J Anim Sci. (2018) 96:3684–98. 10.1093/jas/sky264 30060077 PMC6127775

[19] Svihus B Hervik AK. Digestion and metabolic fates of starch, and its relation to major nutrition–related health problems: a review. Starch-Starke. (2016) 68:302–13. 10.1002/star.20150029525855820

[20] Rankovic A Adolphe JL Verbrugghe A. Role of carbohydrates in the health of dogs. J Am Vet Med Assoc. (2019) 255:546–54. 10.2460/javma.255.5.546 31429654

[21] Riccardi G Clemente G Giacco R. Glycemic index of local foods and diets: the Mediterranean experience. Nutr Rev. (2003) 61:S56–60. 10.1301/nr.2003.may.S56-S60 12828193

[22] Khan AR Fiaz H. Diabetes mellitus and dietary starch in perspective of blood glycaemic control. J Pak Med Assoc. (2020) 70:1232–9. 10.5455/JPMA.25265 32799279

[23] Fried SK Rao SP. Sugars, hypertriglyceridemia, and cardiovascular disease. Am J Clin Nutr. (2003) 78:873S−80S. 10.1093/ajcn/78.4.873S 14522752

[24] Zhang A Sun H Wang X. Power of metabolomics in biomarker discovery and mining mechanisms of obesity. Obes Rev. (2013) 14:344–9. 10.1111/obr.12011 23279162

[25] Calvani R Brasili E Pratico G Sciubba F Roselli M Finamore A. Application of NMR-based metabolomics to the study of gut microbiota in obesity. J Clin Gastroenterol. (2014) 48:S5–7. 10.1097/MCG.0000000000000236 25291128

[26] Forster GM Stockman J Noyes N Heuberger AL Broeckling CD Bantle CM. A comparative study of serum biochemistry, metabolome and microbiome parameters of clinically healthy, normal weight, overweight, and obese companion dogs. Top Companion Anim Med. (2018) 33:126–35. 10.1053/j.tcam.2018.08.003 30502863

[27] Ephraim E Cochrane CY Jewell DE. Varying protein levels influence metabolomics and the gut microbiome in healthy adult dogs. Toxins. (2020) 12:517. 10.3390/toxins12080517 32806674 PMC7472411

[28] Ferrier L Robert P Dumon H Martin L Nguyen P. Evaluation of body composition in dogs by isotopic dilution using a low-cost technique, Fourier-transform infrared spectroscopy. J Nutr. (2002) 132:1725S−7S. 10.1093/jn/132.6.1725S 12042507

[29] Prosky L Asp NG Schweizer TF DeVries JW Furda I. Determination of insoluble, soluble, and total dietary fiber in foods and food products: interlaboratory study. J Assoc Off Anal Chem. (1988) 71:1017–23. 10.1093/jaoac/71.5.1017 2853153

[30] German A Hervera M Hunter L Holden S Morris P Biourge V. Improvement in insulin resistance and reduction in plasma inflammatory adipokines after weight loss in obese dogs. Domest Anim Endocrinol. (2009) 37:214–26. 10.1016/j.domaniend.2009.07.001 19674864

[31] Vreken P van Lint A Bootsma A Overmars H Wanders R van Gennip A. Rapid diagnosis of organic acidemias and fatty-acid oxidation defects by quantitative electrospray tandem-MS acyl-carnitine analysis in plasma. Adv Exp Med Biol. (1999) 466:327–37. 10.1007/0-306-46818-2_38 10709660

[32] Rizzo C Boenzi S Wanders R Duran M Caruso U Dionisi-Vici C. Characteristic acylcarnitine profiles in inherited defects of peroxisome biogenesis: a novel tool for screening diagnosis using tandem mass spectrometry. Pediatr Res. (2003) 53:1013–8. 10.1203/01.PDR.0000064902.59052.0F 12646728

[33] Heilmann RM Cranford SM Ambrus A Grutzner N Schellenberg S Ruaux CG. Validation of an enzyme-linked immunosorbent assay (ELISA) for the measurement of canine S100A12. Vet Clin Pathol. (2016) 45:135–47. 10.1111/vcp.12320 26765807

[34] Castro-Montoya J de Campeneere S van Ranst G Fievez V. Interactions between methane mitigation additives and basal substrates on in vitro methane and VFA production. Anim Feed Sci Technol. (2012) 176:47–60. 10.1016/j.anifeedsci.2012.07.007

[35] Chaney AL Marbach EP. Modified reagents for determination of urea and ammonia. Clin Chem. (1962) 8:130–2. 10.1093/clinchem/8.2.130 13878063

[36] Peters IR Helps CR Calvert EL Hall EJ. Day MJ Identification of four allelic variants of the dog IGHA gene. Immunogenetics. (2004) 56:254–60. 10.1007/s00251-004-0686-x 15241634

[37] Mercier E Peters IR Day MJ Clercx C Peeters D. Toll-and NOD-like receptor mRNA expression in canine sino-nasal aspergillosis and idiopathic lymphoplasmacytic rhinitis. Vet Immunol Immunopathol. (2012) 145:618–24. 10.1016/j.vetimm.2012.01.009 22321737

[38] Vandesompele J de Preter K Pattyn F Poppe B van Roy N de Paepe A. Accurate normalization of real-time quantitative RT-PCR data by geometric averaging of multiple internal control genes. Genome Biol. (2002) 3:1–2. 10.1186/gb-2002-3-7-research0034 12184808 PMC126239

[39] Vanden Bussche J Marzorati M Laukens D Vanhaecke L. Validated high resolution mass spectrometry-based approach for metabolomic fingerprinting of the human gut phenotype. Anal Chem. (2015) 87:10927–10934. 10.1021/acs.analchem.5b02688 26451617

[40] de Paepe E van Meulebroek L Rombouts C Huysman S Verplanken K Lapauw B. A validated multi-matrix platform for metabolomic fingerprinting of human urine, feces and plasma using ultra-high performance liquid-chromatography coupled to hybrid orbitrap high-resolution mass spectrometry. Anal Chim Acta. (2018) 1033:108–18. 10.1016/j.aca.2018.06.065 30172316

[41] Sumner LW Amberg A Barrett D Beale MH Beger R Daykin CA. Proposed minimum reporting standards for chemical analysis Chemical Analysis Working Group (CAWG) Metabolomics Standards Initiative (MSI). Metabolomics. (2007) 3:211–21. 10.1007/s11306-007-0082-2 24039616 PMC3772505

[42] Deng P Swanson KS. Gut microbiota of humans, dogs and cats: current knowledge and future opportunities and challenges. Br J Nutr. (2015) 113:S6–17. 10.1017/S0007114514002943 25414978

[43] Wambacq W Rybachuk G Jeusette I Rochus K Wuyts B Fievez V Hesta M. Fermentable soluble fibres spare amino acids in healthy dogs fed a low-protein diet. BMC Vet Res. (2016) 12:1–10. 10.1186/s12917-016-0752-2 27353524 PMC4924337

[44] Hervera M Castrillo C Albanell E Baucells MD. Use of near-infrared spectroscopy to predict energy content of commercial dog food. J Anim Sci. (2012) 90:4401–7. 10.2527/jas.2012-5106 23100585

[45] Kimura T. The regulatory effects of resistant starch on glycaemic response in obese dogs. Arch Anim Nutr. (2013) 67:503–9. 10.1080/1745039X.2013.857081 24228912

[46] Forsythe LK Wallace JM Livingstone MBE. Obesity and inflammation: the effects of weight loss. Nutr Res Rev. (2008) 21:117–33. 10.1017/S0954422408138732 19087366

[47] Kim KA Gu W Lee IA Joh EH Kim DH. High fat diet-induced gut microbiota exacerbates inflammation and obesity in mice via the TLR4 signaling pathway. PloS one. (2012) 7:e47713. 10.1371/journal.pone.0047713 23091640 PMC3473013

[48] Waise TZ Toshinai K Naznin F NamKoong C Moin ASM Sakoda H. One-day high-fat diet induces inflammation in the nodose ganglion and hypothalamus of mice. Biochem Biophys Res Commun. (2015) 464:1157–62. 10.1016/j.bbrc.2015.07.097 26208455

[49] de la Serre CB Ellis CL Lee J Hartman AL Rutledge JC Raybould HE. Propensity to high-fat diet-induced obesity in rats is associated with changes in the gut microbiota and gut inflammation. Am J Physiol Gastrointest Liver Physiol. (2010) 299:G440–8. 10.1152/ajpgi.00098.2010 20508158 PMC2928532

[50] Lee YS Li P Huh JY Hwang IJ Lu M Kim JI. Inflammation is necessary for long-term but not short-term high-fat diet–induced insulin resistance. Diabetes. (2011) 60:2474–83. 10.2337/db11-0194 21911747 PMC3178297

[51] National Research Council. Nutrient Requirements of Dogs and Cats. Washington, DC: National Academies Press (2006).

[52] Lutz TA Woods SC. Overview of animal models of obesity. Curr Protoc Pharmacol. (2012) 5:61. 10.1002/0471141755.ph0561s58 22948848 PMC3482633

[53] Hewson-Hughes AK Gilham MS Upton S Colyer A Butterwick R Miller AT. The effect of dietary starch level on postprandial glucose and insulin concentrations in cats and dogs. Br J Nutr. (2011) 106:S105–9. 10.1017/S0007114511001887 22005401

[54] Chen XM Zhang WQ Tian Y Wang LF Chen CC Qiu CM. Liraglutide suppresses non-esterified free fatty acids and soluble vascular cell adhesion molecule-1 compared with metformin in patients with recent-onset type 2 diabetes. Cardiovasc Diabetol. (2018) 17:53. 10.1186/s12933-018-0701-4 29636047 PMC5891985

[55] Shen H Lu J Shi TT Cheng C Liu JY Feng JP. Correlation between normal range of serum alanine aminotransferase level and metabolic syndrome: a community-based study. Medicine. (2018) 97:e12767. 10.1097/MD.0000000000012767 30313088 PMC6203538

[56] Lawrence YA Guard BC Steiner JM Suchodolski JS Lidbury JA. Untargeted metabolomic profiling of urine from healthy dogs and dogs with chronic hepatic disease. PloS one. (2019) 14:e0217797. 10.1371/journal.pone.0217797 31150490 PMC6544284

[57] O'Kell AL Garrett TJ Wasserfall C Atkinson MA. Untargeted metabolomic analysis in non-fasted diabetic dogs by UHPLC–HRMS. Metabolomics. (2019) 15:15. 10.1007/s11306-019-1477-6 30830416 PMC6461041

[58] Jackson MI Jewell DE. Soluble and insoluble fiber differentially impact canine faecal microbiome and circulating metabolome. FASEB J. (2016) 30:124–6. 10.1096/fasebj.30.1_supplement.124.6

[59] Allaway D Kamlage B Gilham MS Hewson-Hughes AK Wiemer JC Colyer A. Effects of dietary glucose supplementation on the fasted plasma metabolome in cats and dogs. Metabolomics. (2013) 9:1096–108. 10.1007/s11306-013-0527-8

[60] Finkelstein JD. Methionine metabolism in mammals. J Nutr Biochem. (1990) 1:228–37. 10.1016/0955-2863(90)90070-2 15539209

[61] Yin J Ren W Yang G Duan J Huang X Fang R. l-Cysteine metabolism and its nutritional implications. Mol Nutr Food Res. (2016) 60:134–46. 10.1002/mnfr.201500031 25929483

[62] Martinez Y Li X Liu G Bin P Yan W Mas D. The role of methionine on metabolism, oxidative stress, and diseases. Amino Acids. (2017) 49:2091–8. 10.1007/s00726-017-2494-2 28929442

[63] Carneiro-Filho BA Bushen OY Brito GAC Lima AAM Guerrant RL. Glutamine analogues as adjunctive therapy for infectious diarrhea. Curr Infect Dis Rep. (2003) 5:114–9. 10.1007/s11908-003-0046-2 12641996

[64] Xu W Lin L Liu A Zhang T Zhang S Li Y. L-Theanine affects intestinal mucosal immunity by regulating short-chain fatty acid metabolism under dietary fiber feeding. Food Funct. (2020) 11:8369–79. 10.1039/D0FO01069C 32935679

[65] Zhang C Wang C Chen K Zhao X Geng Z. Effect of l-theanine on growth performance, intestinal development and health, and peptide and amino acid transporters expression of broilers. J Sci Food Agric. (2020) 100:1718–25. 10.1002/jsfa.10192 31821574

[66] Caldovic L Tuchman M. N-acetylglutamate and its changing role through evolution. Biochem J. (2003) 372:279–90. 10.1042/bj20030002 12633501 PMC1223426

[67] Newsholme P Procopio J Lima MMR Pithon-Curi TC Curi R. Glutamine and glutamate-their central role in cell metabolism and function. Cell Biochem Funct. (2003) 21:1–9. 10.1002/cbf.1003 12579515

[68] Dallas DC Sanctuary MR Qu Y Khajavi SH van Zandt AE Dyandra M. Personalizing protein nourishment. Crit Rev Food Sci Nutr. (2017) 57:3313–31. 10.1080/10408398.2015.1117412 26713355 PMC4927412

[69] Nakamura A Ooga T Matsumoto M. Intestinal luminal putrescine is produced by collective biosynthetic pathways of the commensal microbiome. Gut Microbes. (2019) 10:159–71. 10.1080/19490976.2018.1494466 30183487 PMC6546329

[70] Pegg AE. Mammalian polyamine metabolism and function. IUBMB Life. (2009) 61:880–94 10.1002/iub.230 19603518 PMC2753421

[71] Zhang M Caragine T Wang H Cohen PS Botchkina G Soda K. Spermine inhibits proinflammatory cytokine synthesis in human mononuclear cells: a counterregulatory mechanism that restrains the immune response. J Exp Med. (1997) 185:1759–68 10.1084/jem.185.10.1759 9151701 PMC2196317

[72] Azad MAK Bin P Liu G Fang J Li T Yin Y. Effects of different methionine levels on offspring piglets during late gestation and lactation. Food Funct. (2018) 9:5843–54. 10.1039/C8FO01343H 30358792

[73] Yannai S. Dictionary of food compounds with CD-ROM. In: Additives, Flavors, and Ingredients. New York, NY: CRC Press (2003).

[74] Lampis G Deidda D Maullu C Petruzzelli S Pompei R Monache FD. Karalicin, a new biologically active compound from Pseudomonas fluorescens/putida I Production, isolation, physico-chemical properties and structure elucidation. J Antibiot. (1996) 49:260–2. 10.7164/antibiotics.49.260 8626241

[75] Ulaszewska M Weinert CH Trimigno A Portmann R Andres Lacueva C Badertscher R. Nutrimetabolomics: an integrative action for metabolomic analyses in human nutritional studies. Mol Nutr Food Res. (2019) 63:1800384. 10.1002/mnfr.201970001 30176196

[76] Bland IM Guthrie-Jones A Taylor RD Hill J. Dog obesity: owner attitudes and behaviour. Prev Vet Med. (2009) 92:333–40. 10.1016/j.prevetmed.2009.08.016 19766333

