# Supplementary material for: Differences in Metabolic Profiles of Healthy Dogs Fed a High-Fat vs. a High-Starch Diet
Source: Front Vet Sci. 2022 Feb 17;9:801863. doi: 10.3389/fvets.2022.801863 (PMC8891928; doi:10.3389/fvets.2022.801863)
Supplement: Supplementary file 1 [file Data_Sheet_1.pdf]

**Table S1.** Primers used for qPCR Assays

| Gene | Forward | Reverse |
|---|---|---|
| SDHA | GCCTTGGATCTCTTGATGGA | TTCTTGGCTCTTATGCGATG |
| TBP | CTATTTCTTGGTGTGCATGAGG | CCTCGGCATTCAGTCTTTTC |
| YWHAZ | CGAAGTTGCTGCTGGTGA | TTGCATTTCCTTTTTGCTGA |
| IL-6 | CTCTCCACAAGCGCCTTCTC | TGAAGTGGCATCATCCTTGG |
| IL-8 | CACTCCACACCTTTCCATCC | GTCCAGGCACACCTCATTTC |
| IL-10 | CGACCCAGACATCAAGAACC | CACAGGGAAGAAATCGGTGA |
| IL-18 | TTAAAGCGGAAAGTGATGAAGG | TCGGGCATATCCTCAAATACA |
| TNF-α | CATGTGCTCCTCACCCACAC | AGGGCTCTTGATGGCAGAGA |
| TLR4 | GTGCTTCATGGTTTCTCTGGT | CCAGTCTTCATCCTGGCTTG |
| CD14 | CACACTCGCTTGCTTTTTCC | CGGGATTGTCGGATAGGTC |
| IL-1β | CTGCTGCCAAGACCTGAAC | GACTGACACGAAATGCCTCA |
| IL-1β positive control | CTGATGGCCCTGGAAATG | TGTGGCTTATGTCCTGTAACTTG |
| IL-1α | AGCCTCATGCCGTGTTCTT | CTTGATCCTTGTTCTTACTCAGGTC |
| IL-1α positive control | CCTCCTTTTCCTGTCCCATT | ACTCAAAGCTGGTGGTAGGG |

**Table S2.** Body and faecal parameters (non significant findings, n = 5).

| Item | HS | HF | Period 1 | Period 2 | *p* value | | |
|---|---|---|---|---|---|---|---|
| | | | | | Diet | Period | Diet*Period |
| Body weight (kg) | 10.60 ± 1.22 | 10.29 ± 1.20 | 10.49 ± 1.17 | 10.40 ± 1.29 | 0.721 | 0.789 | 0.817 |
| Body weight (kg; W6) | 10.23 ± 1.22 | 10.77 ± 1.30 | 10.40 ± 1.29 | 10.60 ± 1.28 | 0.775 | 0.674 | 0.618 |
| Fat free mass (%) | 8.32 ± 1.13 | 8.69 ± 0.82 | 8.59 ± 0.82 | 8.43 ± 1.11 | 0.414 | 0.414 | 0.414 |
| Fat mass (%) | 2.17 ± 0.33 | 2.10 ± 0.47 | 2.23 ± 0.31 | 2.06 ± 0.48 | 0.834 | 0.834 | 0.834 |
| Fat (%) | 20.80 ± 2.91 | 19.54 ± 4.58 | 20.72 ± 3.08 | 19.73 ± 4.36 | 0.753 | 0.753 | 0.753 |
| Fat free mass (%; W6) | 7.68 ± 1.13 | 7.70 ± 1.09 | 7.45 ± 1.17 | 7.91 ± 0.99 | 0.826 | 0.983 | 0.817 |
| Fat mass (%; W6) | 2.53 ± 0.46 | 3.07 ± 0.57 | 2.95 ± 0.44 | 2.69 ± 0.65 | 0.760 | 0.244 | 0.350 |
| Fat (%; W6) | 24.90 ± 4.68 | 28.52 ± 4.19 | 28.51 ± 3.54 | 25.26 ± 4.71 | 0.993 | 0.312 | 0.568 |
| Body condition score | 4.00 ± 0.00 | 4.20 ± 0.42 | 4.00 ± 0.00 | 4.20 ± 0.42 | 0.317 | 0.317 | 0.122 |
| Faecal score | 2.23 ± 0.39 | 2.59 ± 0.54 | 2.20 ± 0.22 | 2.70 ± 0.57 | 0.849 | 0.975 | 0.433 |
| Faecal pH | 6.80 ± 0.38 | 7.17 ± 0.13 | 7.10 ± 0.32 | 6.90 ± 0.33 | 0.415 | 0.532 | 0.94 |
| S100A12 (ng/g) | 549.7 ± 682.5 | 3272.4 ± 2636.3 | 1465.1 ± 1900.3 | 2357.2 ± 2735.1 | 0.592 | 0.913 | 0.655 |
| Dry matter (%) | 34.84 ± 8.86 | 42.39 ± 6.45 | 39.60 ± 7.36 | 37.70 ± 9.78 | 0.867 | 0.371 | 0.441 |
| $NH_3$ (mmol/g) | 1465.5 ± 753.6 | 1644.4 ± 645.2 | 1491.7 ± 760.3 | 1618.3 ± 644.2 | 0.177 | 0.191 | 0.218 |
| Acetate (µmol/g) | 277.6 ± 170.1 | 154.0 ± 48.72 | 192.7 ± 67.90 | 238.8 ± 184.4 | 0.703 | 0.222 | 0.302 |
| Propionate (µmol/g) | 80.92 ± 15.46 | 75.25 ± 22.81 | 80.80 ± 23.81 | 75.30 ± 13.91 | 0.947 | 0.952 | 0.894 |
| Iso-butyrate (µmol/g) | 11.33 ± 4.00 | 12.03 ± 2.92 | 11.30 ± 3.77 | 12.10 ± 3.19 | 0.346 | 0.336 | 0.392 |
| Butyrate (µmol/g) | 84.09 ± 43.43 | 37.30 ± 12.42 | 62.40 ± 35.27 | 58.90 ± 44.79 | 0.421 | 0.947 | 0.885 |
| Iso-valerate (µmol/g) | 16.91 ± 5.79 | 7.07 ± 8.09 | 16.70 ± 5.29 | 18.10 ± 4.61 | 0.278 | 0.673 | 0.538 |

**Table S3.** Blood parameters (non significant findings, n = 5)

| Item | HS | HF | Period 1 | Period 2 | *p* value | | |
|---|---|---|---|---|---|---|---|
| | | | | | **Diet** | **Period** | **Diet*Period** |
| FG (µM) | 164.2 ± 38.8 | 155.4 ± 40.3 | 154.8 ± 41.7 | 164.8 ± 37.04 | 0.819 | 0.610 | 0.711 |
| CHOL (µM) | 178.0 ± 35.1 | 164.7 ± 33.7 | 170.3 ± 39.0 | 172.4 ± 30.7 | 0.147 | 0.221 | 0.208 |
| TG (µM) | 39.70 ± 16.06 | 27.90 ± 8.43 | 31.10 ± 10.77 | 36.50 ± 16.55 | 0.512 | 0.757 | 0.924 |
| GLU (µM) | 78.40 ± 6.28 | 85.70 ± 5.21 | 83.10 ± 5.99 | 81.00 ± 7.60 | 0.054 | 0.324 | 0.134 |
| INS (µM) | 12.92 ± 5.11 | 39.20 ±48.48 | 37.72 ± 49.10 | 14.40 ± 6.93 | 0.870 | 0.892 | 0.762 |
| GLC/INS | 16.25 ± 5.54 | 46.46 ± 59.15 | 45.24 ± 59.59 | 17.48 ± 7.53 | 0.063 | 0.036 | 0.145 |
| Gly (µM) | 190.1 ± 24.12 | 186.9 ± 37.32 | 201.0 ± 33.47 | 176.0 ± 22.61 | 0.094 | 0.220 | 0.077 |
| Ala (µM) | 358.6 ± 118.5 | 239.5 ±44.27 | 335.4 ± 129.1 | 262.8 ± 65.54 | 0.089 | 0.159 | 0.347 |
| Val (µM) | 534.7 ± 163.2 | 526.1 ± 170.6 | 518.6 ± 183.1 | 542.2 ± 148.1 | 0.492 | 0.567 | 0.498 |
| Leu (µM) | 201.1 ± 31.06 | 179.8 ±43.47 | 182.8 ± 32.78 | 198.1 ± 43.60 | 0.843 | 0.470 | 0.610 |
| Orn (µM) | 17.83 ± 4.30 | 15.63 ± 2.26 | 16.67 ± 3.63 | 16.79 ± 3.62 | 0.545 | 0.826 | 0.806 |
| Met (µM) | 60.11 ± 8.67 | 58.90 ± 9.22 | 59.91 ± 5.66 | 59.10 ± 11.34 | 0.983 | 0.964 | 0.925 |
| Phe (µM) | 85.11 ± 18.94 | 84.04 ± 16.98 | 80.64 ± 11.61 | 88.51 ± 21.88 | 0.139 | 0.077 | 0.120 |
| Cit (µM) | 30.82 ± 10.29 | 19.07 ± 6.74 | 23.96 ± 10.24 | 25.93 ± 11.01 | 0.185 | 0.603 | 0.520 |
| Tyr (µM) | 54.87 ±8.93 | 51.45 ± 10.59 | 53.82 ± 8.63 | 52.49 ± 11.09 | 0.113 | 0.085 | 0.058 |
| C0 (µM) | 47.51 ± 10.59 | 45.39 ± 9.75 | 45.80 ± 10.14 | 47.09 ± 10.29 | 0.708 | 0.836 | 0.786 |
| C2 (µM) | 8.97 ± 1.96 | 10.12 ± 1.83 | 9.14 ± 2.01 | 9.94 ± 1.88 | 0.683 | 0.763 | 0.955 |
| C3 (µM) | 0.279 ± 0.069 | 0.334 ± 0.125 | 0.273 ± 0.097 | 0.340 ± 0.101 | 0.853 | 0.908 | 0.611 |
| C4 (µM) | 0.175 ± 0.054 | 0.164 ± 0.043 | 0.174 ± 0.050 | 0.165 ± 0.048 | 0.113 | 0.118 | 0.134 |
| C5 (µM) | 0.249 ± 0.093 | 0.259 ± 0.069 | 0.255 ± 0.095 | 0.253 ± 0.067 | 0.616 | 0.561 | 0.564 |
| C5:1 (µM) | 0.033 ± 0.013 | 0.031 ± 0.012 | 0.032 ± 0.015 | 0.026 ± 0.011 | 0.731 | 0.796 | 1.000 |
| 3OH-C4 (µM) | 0.027 ± 0.007 | 0.031 ± 0.009 | 0.027 ± 0.009 | 0.031 ± 0.006 | 0.463 | 0.463 | 0.653 |
| 3OH-C5 (µM) | 0.039 ± 0.012 | 0.037 ± 0.013 | 0.035 ± 0.012 | 0.041 ± 0.012 | 0.437 | 0.737 | 0.483 |
| C3DC (µM) | 0.047 ± 0.027 | 0.045 ± 0.022 | 0.042 ± 0.015 | 0.050 ± 0,031 | 0.659 | 0.825 | 0.688 |
| C4DC (µM) | 0.083 ± 0.022 | 0.073 ± 0.024 | 0.081 ± 0.024 | 0.075 ± 0.023 | 0.745 | 0.676 | 0.575 |
| CD14 (µM) | 1.96 ± 0.64 | 2.47 ±0.77 | 2.44 ± 0.82 | 1.99 ± 0.59 | 0.547 | 0.876 | 0.836 |
| IL-10 | 1.98 ± 0.64 | 1.59 ± 0.43 | 1.79 ± 0.51 | 1.78 ± 0.64 | 0.837 | 0.839 | 0.834 |
| IL-18 | 3.79 ± 1.71 | 4.00 ± 1.85 | 4.21 ± 1.61 | 3.57 ± 1.88 | 0.641 | 0.837 | 0.684 |
| IL-1β | 2.40 ± 1.20 | 2.80 ± 1.05 | 3.08 ± 1.22 | 2.13 ± 0.81 | 0.426 | 0.112 | 0.282 |
| IL-1α | 1.90 ± 0.57 | 2.13 ± 0.64 | 2.20 ± 0.73 | 1.83 ± 0.38 | 0.53 | 0.937 | 0.671 |
| IL-8 | 4.13 ± 2.75 | 4.39 ± 4.19 | 5.80 ± 4.32 | 2.72 ± 1.12 | 0.743 | 0.78 | 0.774 |
| TLR-4 | 2.18 ± 0.91 | 2.61 ± 0.82 | 2.23 ± 0.99 | 2.53 ± 0.76 | 0.51 | 0.586 | 0.704 |
| TNFα | 2.04 ± 0.52 | 1.87 ± 0.73 | 2.03 ± 0.71 | 1.89 ± 0.55 | 0.755 | 0.735 | 0.615 |
| NEFA (µM) | 1.13 ± 0.25 | 0.94 ± 0.23 | 1.13 ± 0.31 | 0.95 ± 0.15 | 0.041 | 0.042 | 0.108 |
| LPS (µM) | 0.83 ± 0.19 | 0.96 ± 0.67 | 1.01 ± 0.68 | 0.78 ± 0.09 | 0.378 | 0.668 | 0.455 |
| S100A12 (µg/L) | 64.10 ± 20.65 | 72.60 ± 40.01 | 76.20 ± 38.20 | 60.50 ± 21.70 | 0.128 | 0.311 | 0.157 |

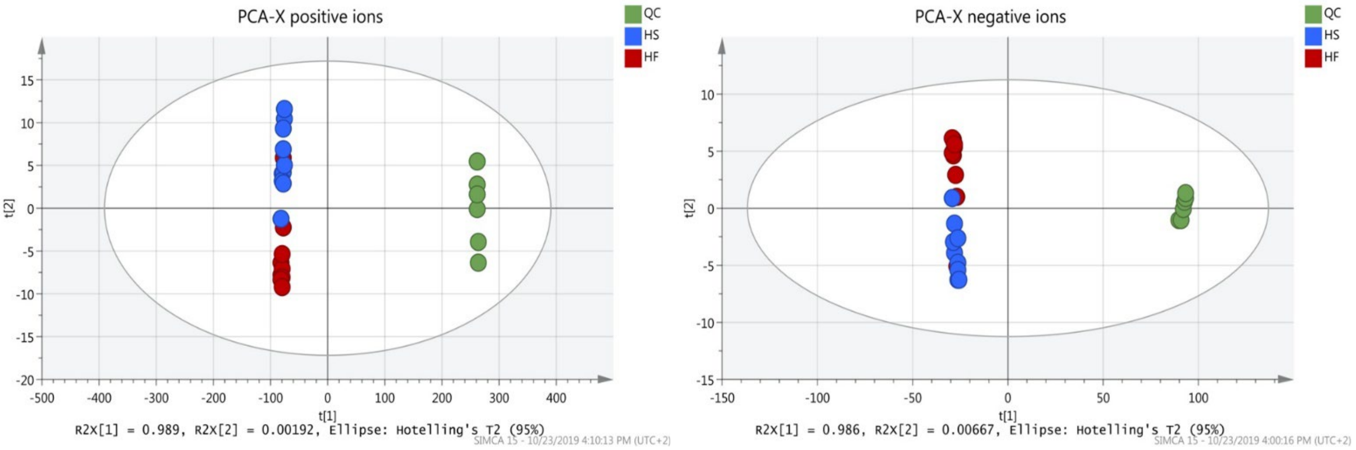


**Figure S1.** PCA-X plot in positive (left) and negative (right) ionization (QC: Quality Control; HS: high-starch diet; HF: high-fat diet).

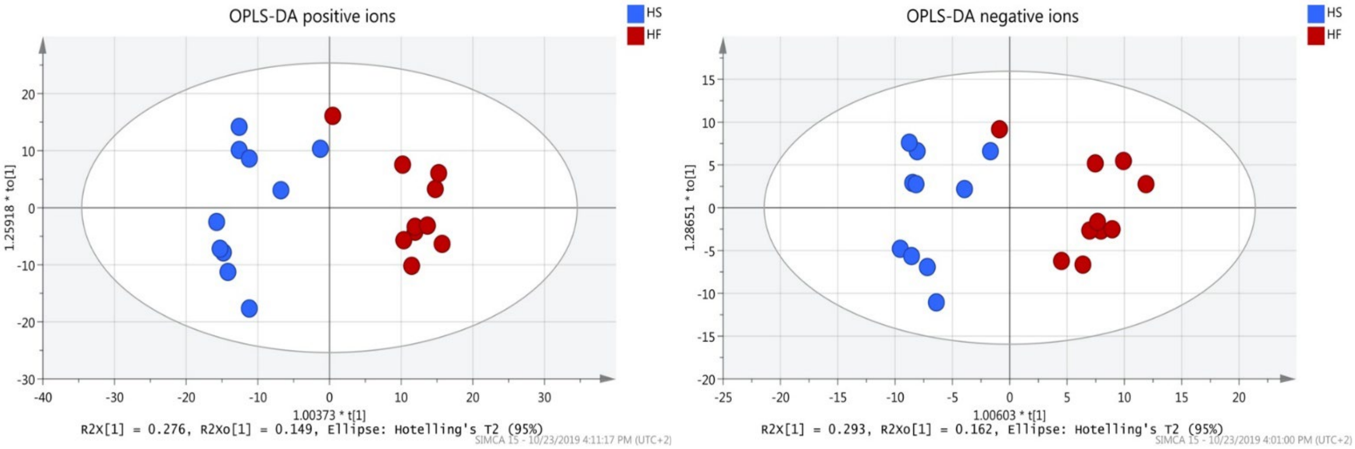


**Figure S2.** OPLS-DA plot in positive (left) and negative (right) ionization (HS: high-starch diet; HF: high-fat diet).

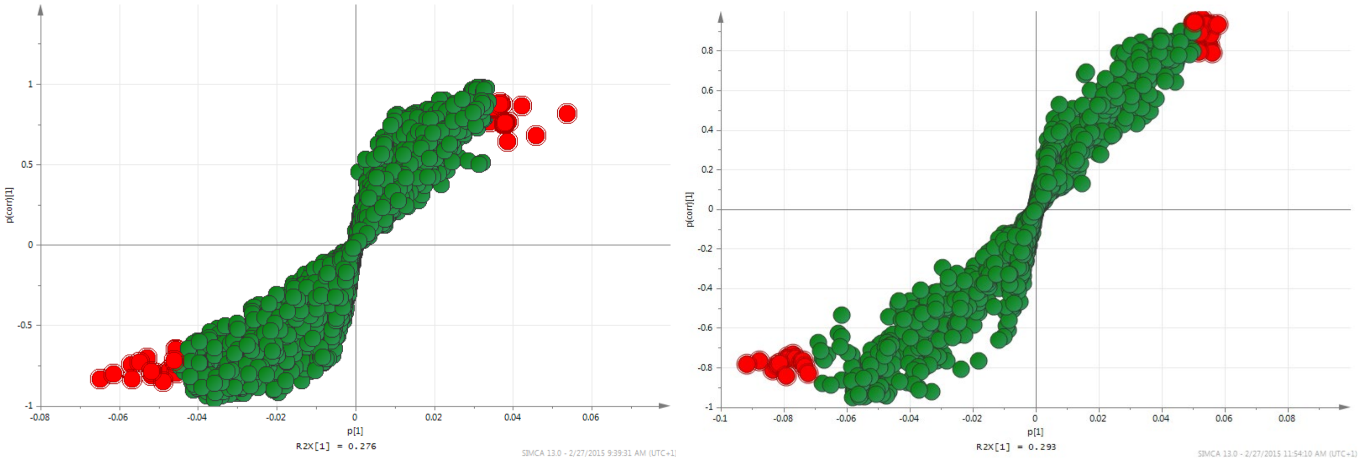


**Figure S3.** S-plot (and selection of eccentric metabolites) of OPLS-DA models based on detected positive (left) and negative (right) ions.

**Table S4.** Results of two-way ANOVA for effect of diet, period, and interaction for the retrieved discriminative metabolites (n = 5).

| **Metabolites** | ***P* value** | | |
| --- | --- | --- | --- |
| | **Diet** | **Period** | **Diet*Period** |
| Unidentified_1 | 0.012 | 0.735 | 0.047 |
| Unidentified_4 | 0.002 | 0.735 | 0.218 |
| Unidentified_2 | 0.046 | 0.735 | 0.218 |
| L-Lysopine/(Iso)leucyl-serine/seryl-(Iso)leucine/Valyl-Threonine/Threoninyl-Valine | 0.001 | 0.787 | 0.221 |
| Unidentified_5 | 0.001 | 0.566 | 0.221 |
| Unidentified_3 | 0.003 | 0.139 | 0.221 |
| (Iso)leucyl-valine/Valyl-(Iso)leucine | 0.001 | 0.787 | 0.226 |
| (Iso)leucyl-(Iso)leucine | 0.002 | 0.735 | 0.226 |
| Spermic acid 2/(Iso)leucyl-threonine/ Threoninyl-(Iso)leucine | 0.003 | 0.735 | 0.226 |
| Spermic acid 2/(Iso)leucyl-threonine/ Threoninyl-(Iso)leucine | 0.002 | 0.787 | 0.357 |
| Valyl-valine | 0.001 | 0.735 | 0.582 |
| (Iso)leucyl-Threoninyl-Valine | 0.015 | 0.566 | 0.610 |
| Spermic acid 2/(Iso)leucyl-threonine/ Threoninyl-(Iso)leucine | 0.001 | 0.735 | 0.689 |
| Glycyl-Valine/Glycine-Norvaline/Valyl-Glycine/L-Theanine/N-acetylornithine | 0.002 | 0.787 | 0.970 |

**Table S5.** IUPAC names of putatively annotated metabolites

| Name | IUPAC name |
|---|---|
| L-Methionine | (2S)-2-amino-4-(methylsulfanyl)butanoic acid |
| Glycyl-Valine | (2S)-2-(2-aminoacetamido)-3-methylbutanoic acid |
| Glycyl-Norvaline | 2-[(2-amino-1-hydroxyethylidene)amino]pentanoic acid |
| Valyl-Glycine | 2-[(2S)-2-amino-3-methylbutanamido]acetic acid |
| N2-acetylornithine | (2S)-5-amino-2-acetamidopentanoic acid |
| N5-acetylornithine | (2S)-2-amino-5-acetamidopentanoic acid |
| spermic acid 2 | 3-[4-(2-carboxyethylamino)butylamino]propanoic acid |
| Leucyl-Threonine | 2-(2-amino-4-methylpentanamido)-3-hydroxybutanoic acid |
| Isoleucyl-Threonine | 2-(2-amino-3-methylpentanamido)-3-hydroxybutanoic acid |
| Threonyl-Leucine | (2S)-2-[(2S,3R)-2-amino-3-hydroxybutanamido]-4-methylpentanoic acid |
| Threonyl-Isoleucine | (2S,3S)-2-[(2S,3R)-2-amino-3-hydroxybutanamido]-3-methylpentanoic acid |
| L-Lysopine | 6-amino-2-[(1-carboxyethyl)amino]hexanoic acid |
| Leucyl-Serine | 2-[(2-amino-1-hydroxy-4-methylpentylidene)amino]-3-hydroxypropanoic acid |
| Isoleucyl-Serine | 2-(2-amino-3-methylpentanamido)-3-hydroxypropanoic acid |
| Seryl-Isoleucine | (2S,3S)-2-[(2S)-2-amino-3-hydroxypropanamido]-3-methylpentanoic acid |
| Valyl-Threonine | (2S)-2-[(2S)-2-amino-3-methylbutanamido]-3-hydroxybutanoic acid |
| Threoninyl-Valine | (2S)-2-[(2S,3R)-2-amino-3-hydroxybutanamido]-3-methylbutanoic acid |
| Valyl-valine | (2S)-2-[(2S)-2-amino-3-methylbutanamido]-3-methylbutanoic acid |
| Leucyl-Threonine | 2-(2-amino-4-methylpentanamido)-3-hydroxybutanoic acid |
| Isoleucyl-Threonine | 2-(2-amino-3-methylpentanamido)-3-hydroxybutanoic acid |
| Threoninyl-Isoleucine | (2S,3S)-2-[(2S,3R)-2-amino-3-hydroxybutanamido]-3-methylpentanoic acid |
| Threoninyl-Leucine | (2S)-2-[(2S,3R)-2-amino-3-hydroxybutanamido]-4-methylpentanoic acid |
| Leucyl-Threonine | 2-(2-amino-4-methylpentanamido)-3-hydroxybutanoic acid |
| Isoleucyl-Threonine | 2-(2-amino-3-methylpentanamido)-3-hydroxybutanoic acid |
| Threoninyl-Isoleucine | (2S,3S)-2-[(2S,3R)-2-amino-3-hydroxybutanamido]-3-methylpentanoic acid |
| Threoninyl-leucine | (2S)-2-[(2S,3R)-2-amino-3-hydroxybutanamido]-4-methylpentanoic acid |
| Leucyl-Valine | 2-(2-amino-4-methylpentanamido)-3-methylbutanoic acid |
| Isoleucyl-Valine | 2-(2-amino-3-methylpentanamido)-3-methylbutanoic acid |
| Valyl-Isoleucine | (2S,3S)-2-[(2S)-2-amino-3-methylbutanamido]-3-methylpentanoic acid |
| Valyl-Leucine | (2S)-2-[(2S)-2-amino-3-methylbutanamido]-4-methylpentanoic acid |
| Leucyl-Threoninyl-Valine | (2S)-2-[[(2S,3R)-2-[[(2S)-2-amino-4-methylpentanoyl]amino]-3-hydroxybutanoyl]amino]-3-methylbutanoic acid |
| Isoleucyl-Threoninyl-Valine | (2S)-2-[[(2S,3R)-2-[[(2S,3S)-2-amino-3-methylpentanoyl]amino]-3-hydroxybutanoyl]amino]-3-methylbutanoic acid |
| Leucyl-Isoleucine | 2-(2-amino-4-methylpentanamido)-3-methylpentanoic acid |
| Leucyl-Leucine | (2S)-2-[(2S)-2-amino-4-methylpentanamido]-4-methylpentanoic acid |
| Isoleucyl-Leucine | 2-(2-amino-3-methylpentanamido)-4-methylpentanoic acid |
